# Heinz body hemolysis following extended use phenazopyridine in a post‐HCT patient with hemorrhagic cystitis: An old problem for a new generation

**DOI:** 10.1002/ccr3.7012

**Published:** 2023-03-07

**Authors:** Asmaa Ferdjallah, Susie Long, Vanessa Dayton, Ashish O. Gupta

**Affiliations:** ^1^ Division of Blood and Marrow Transplant and Cellular Therapy, Department of Pediatrics University of Minnesota Minneapolis Minnesota USA; ^2^ MHealth Fairview, Acute Care Pharmacy Services Minneapolis Minnesota USA; ^3^ Department of Pathology, Hematopathology University of Minnesota Minneapolis Minnesota USA

**Keywords:** Heinz body, hemolysis, hemorrhagic cystitis, pediatrics, phenazopyridine

## Abstract

Hemorrhagic cystitis is a common complication following the use of cyclophosphamide. Associated dysuria can be painful and there are few good options to relieve pain. Phenazopyridine has historically been utilized for dysuria and is available over the counter. However, it is associated with hematologic side effects with prolonged use. Here we present a case of a patient who developed Heinz body hemolysis following prolonged administration of phenazopyridine to treat cyclophosphamide‐induced hemorrhagic cystitis following hematopoietic stem cell transplant.

## Graphical abstract

Phenazopyridine is an over‐the‐counter medication used for hemorrhagic cystitis symptoms. We present a case of Heinz body hemolysis following prolonged administration of phenazopyridine to treat cyclophosphamide‐induced hemorrhagic cystitis following hematopoietic stem cell transplant. Prolonged phenazopyridine use should be carefully monitored for hematologic side effects.

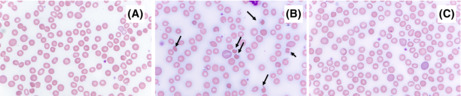

## 1 INTRODUCTION

Hemorrhagic cystitis (HC) is a common complication following hematopoietic stem cell transplant (HCT) associated with viral infections and/or administration of high dose cyclophosphamide (CY).^1^, ^2^ Preventative measures including hyperhydration and the acrolein‐neutralizing agent mesna are useful in preventing HC but do not prevent all cases.^3^ Once HC occurs, the associated dysuria can be debilitating and difficult to treat.^1^ For this reason, multiple agents are used to treat severe dysuria. This poly‐pharmacy approach may lead to drug‐related adverse effects and complicate the post‐HCT recovery phase. We present a case of a girl who experienced HC secondary to CY, treated conservatively with hyperhydration, phenazopyridine, and oxybutynin, who suddenly developed acute hemolysis.

## 2 CASE DESCRIPTION

A 14‐year‐old Caucasian girl with late relapse pre‐B‐cell ALL with amplified RUNX1 (iAMP21 mutation) developed sudden hyperbilirubinemia 28 days following a matched unrelated umbilical cord transplant (UCBT). Prior to receiving her UCBT, she underwent extensive chemotherapy (including inotuzumab ozogamicin and vincristine, among others) and chimeric antigen receptor T cell (CAR‐T) therapy leading to a diagnosis of complex regional pain syndrome. She had no evidence of disease on +25 days post‐HCT bone marrow evaluation and remained 100% donor in her bone marrow by chimerism. She had undergone chemotherapy conditioning with CY, fludarabine, and total body irradiation with post‐HCT complications including persistent nausea, mucositis, presumed fungal pneumonia treated with voriconazole and micafungin, breakthrough menstrual bleeding (despite leuprolide acetate depot prior to conditioning therapy) managed with estradiol daily and dysuria and frequency concerning for HC. Urine studies revealed BK viruria and serum studies were positive but not quantifiable for viremia. Although her nausea and mucositis largely resolved with count recovery on day 21, she continued to experience lingering dysuria and hematuria with small clots concerning for persistent HC. She received additional fluids with phenazopyridine (every 6 h), oxybutynin (every 6 h), and oxycodone for symptomatic control of dysuria.

**FIGURE 1:**
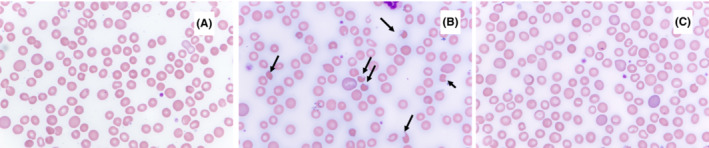
(A) Blood smear on day 19 of phenazopyridine, one day post red cell transfusion. Wrights Giemsa stain, 100× (B) Blood smear on day 25 of phenazopyridine, 6 days post red cell transfusion. Arrows indicate “bite,” “blister,” and “apple core” red cells. Wrights Giemsa stain 100× (C) Blood smear 10 days after phenazopyridine discontinued, 10 days post red cell transfusion. Wrights Giemsa stain 100×.

About 2 weeks after the onset of HC, total bilirubin was noted to be 5.5 mg/dL (normal 0.2–1.3 mg/dL) with a direct bilirubin of 4.4 mg/dL (normal 0.0–0.2 mg/dL) on routine outpatient follow‐up. She underwent an abdominal ultrasound which revealed cholelithiasis without evidence of cholecystitis and hepatomegaly with normal grayscale and doppler evaluation. Her hemoglobin was stable at 9.7 g/dL (normal 11.7–15.7 g/dL) from 10 g/dL the week prior following a red blood cell transfusion. A peripheral blood smear revealed slight normochromic normocytic anemia with red cell regeneration, rare spherocytes (consistent with history of recent red blood cell transfusion), and rare “bite” cells raising the possibility of oxidant hemolysis (Figure 1A). An LDH was elevated at 379 U/L. A direct Coomb's test was negative. The differential diagnosis based on the peripheral blood smear included G6PD deficiency, severe liver disease, and congenital unstable hemoglobin. Clinical considerations with elevated direct hyperbilirubinemia included post‐transplant sinusoidal obstruction syndrome, cholelithiasis, and transient TPN‐induced hyperbilirubinemia. She had normal coagulation markers (INR, PTT, fibrinogen). As a precaution given cholecystitis, she was restarted on ursodiol at 10 mg/kg/daily. Further testing was negative for above mentioned differential.

One week later (post HCT day 34), she continued to have persistent but indirect hyperbilirubinemia (total bilirubin 2.3 mg/dL, indirect bilirubin 2.1 mg/dL), increasing absolute reticulocyte count (195.8 × 10^9^/L) and now rapidly declining hemoglobin (7.6 g/dL) requiring 2 units of red blood cell transfusion. The LDH was 341 U/L. The differential included transplant‐associated thrombotic microangiopathy (TA‐TMA), immune mediated cytopenia, medication induced hemolysis (she was also on sulfamethoxazole/trimethoprim which was started a week prior), or evolving acute graft versus host disease (aGVHD). A repeat peripheral blood smear obtained prior to the transfusion was reported as showing overall normocytic marked anemia with “bite,” “veil,” and “apple core” red cells along with morphologic evidence of increased red cell regeneration consistent with Heinz body hemolysis related to phenazopyridine (Figure 1B). At this point, the patient had been utilizing phenazopyridine at a dose of 200 mg PO QID for 34 days consecutively (cumulative dose 28 g) and was thus discontinued.

**FIGURE 2:**
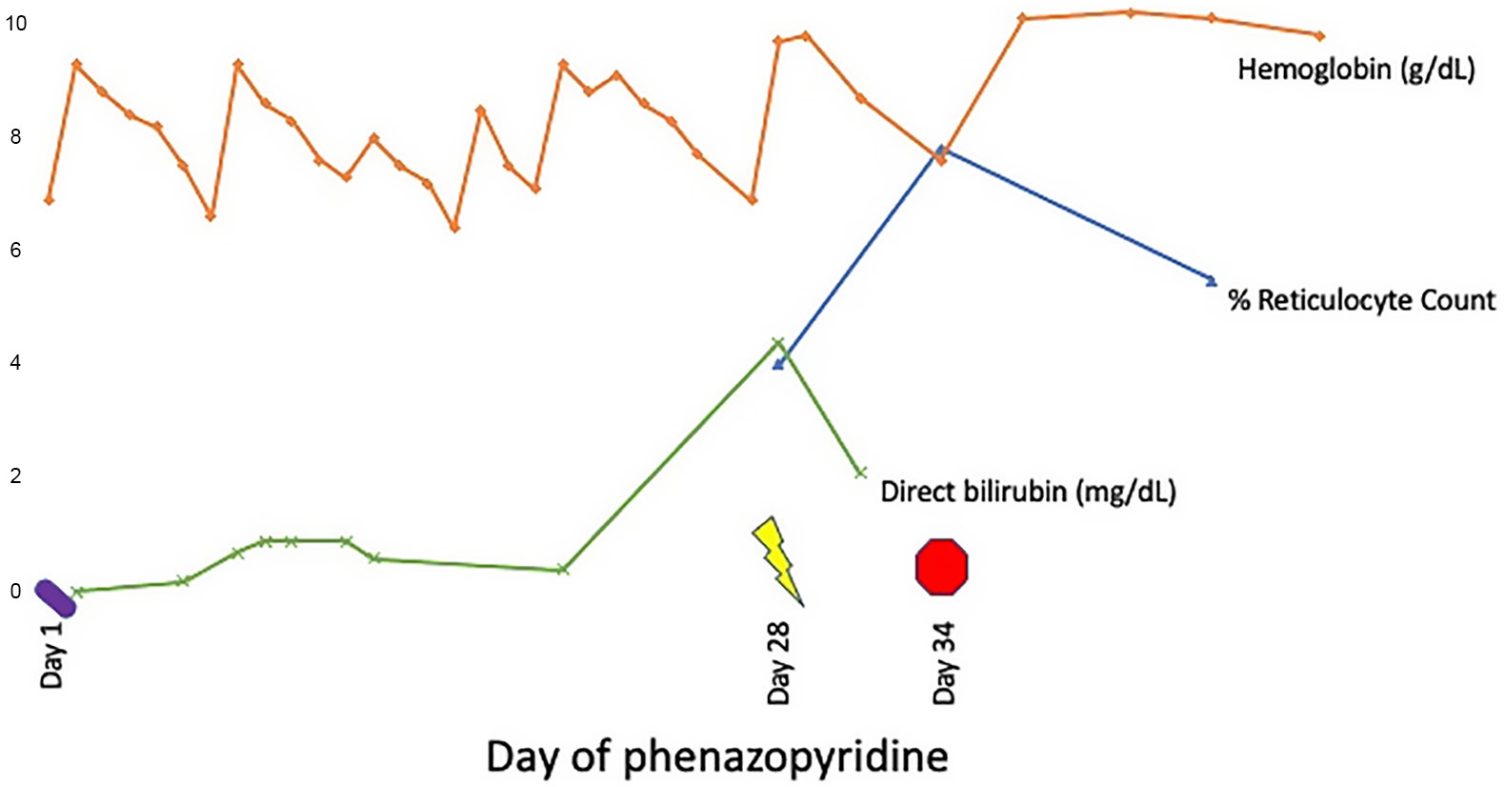
Patient hemoglobin (orange, g/dL), percent reticulocyte count (blue), and direct bilirubin (green, mg/dL) over time. X‐axis describes numerical lab value. Y‐axis describes day of phenazopyridine administration. Day 28 represents day hemolysis noted (yellow lightning bolt) and day 34 represents discontinuation of phenazopyridine.

Ten days later (post‐HCT day 44), a repeat peripheral blood smear showed minimal residual morphologic evidence of Heinz body hemolysis. Ursodiol was discontinued, hemoglobin improved to 10.1 g/dL, total bilirubin was normal at 1.1 mg/dL, absolute reticulocyte count decreased to 175.2 × 10^9^/L, and lactate dehydrogenase decreased to 256 U/L. On last review, the patient continues to do well and has not required further red blood cell transfusions. Table 1 and Figure 2 summarizes the patient's course and lab findings.

**TABLE 1:** Laboratory findings showing rapid onset anemia and recovery after discontinuation of phenazopyridine.

	Phenazopyridine						Phenazopyridine discontinuation			
Day	14	15	16	17	18	Phenazopyridine discontinued	0	3	7	10
WBC	4.2	6.6	4.0	5.7	6.8		7.8	6.0	6.8	10.9
Hemoglobin	8.6	8.3	7.7	6.9	10.0		7.6	10.1	10.2	10.1
Hematocrit	26.9	25.6	24.4	21.7	30.0		25.1	32.3	31.3	31.5
Platelet Count	37	28	20	26	52		52	68	86	104
RBC Count	2.9	2.86	2.64	2.3	3.36		2.54	3.29	3.2	3.18
MCV	93	90	92	94	89		99	98	98	99
MCH	29.7	29	29.2	30	29.8		29.9	30.7	31.9	31.8
MCHC	32	32.4	31.6	31.8	33.3		30.3	31.3	32.6	32.1
RDW	14.6	14.6	15	16.3	18.1		22.1	21.7	21.1	21

*Note*: Patient received 1 unit of packed red blood cells on day 17 of phenazopyridine. WBC normal range 4.0–11.0 10e3/μL, hemoglobin normal range 11.7–15.7 g/dL, hematocrit normal range 35.0%–47.0%, platelet count 150–450 10e3/μL, RBC count 3.70–5.30 10e6/μL, MCV 77–100 fL, MCH 26.5–33.0 pg, MCHC 31.5–36.5 g/dL, and RDW 10.0%–15.0%.

## 3 DISCUSSION

This case highlights the importance of closely monitoring polypharmacy and avoiding the assumption that over the counter (OTC) medications are benign. This patient underwent HCT with a history of complex regional pain disorder. Therefore, she was very aware of any discomfort she felt and was intuitive and vocal about her desires for pain management. In an effort to respect burgeoning autonomy in a young adolescent, she was encouraged to help manage medication choices. She identified phenazopyridine as the single agent that helped to alleviate her dysuria and in fact asked for it to be removed last in an effort to facilitate an oxycodone wean. Unfortunately, despite phenazopyridine being thought of as a benign agent, she developed a rapid onset of hemolysis about 6 weeks into therapy.

Phenazopyridine is an azo dye analgesic used for symptomatic relief of dysuria that exists in many OTC formulations.^4^ It exerts its action via rapid metabolization, urinary excretion and topical effect.^5^ It is intended for use for a short duration due to a number of possible side effects including rash, renal failure, and hemolytic anemia.^6^ Patients who remain on the medication beyond 15 days should be cautioned of risks.^6^ Phenazopyridine is also thought to mask signs and symptoms of urinary tract infection however in our patient her urinalysis and urine culture was negative prior to initiating the agent.

Phenazopyridine is thought to cause hemolysis by a toxic reaction on red blood cells, producing methemoglobinemia, and Heinz body anemia.^7^ From a hematopathologic standpoint, all causes of hemolytic anemia (except for paroxysmal nocturnal hemoglobinuria) have a characteristic red cell morphology. For Heinz body anemia, an accurate understanding of the clinical setting can lead to a rapid diagnosis.

Reports in the literature of hemolysis following the use of phenazopyridine mostly involve small children or elderly individuals. The first published report dates from 1951 and describes a 3‐year‐old who developed methemoglobinemia and hemolysis following the administration of phenazopyridine.^8^ Interesting, Shore et al. reported no incidence of hemolysis following the use of phenazopyridine for greater than 14 days in a cohort of 90 males utilizing the agent for radiation cystitis.^9^ Currently there is no clinical data for phenazopyridine use for HC following HCT.

Although this patient remained on phenazopyridine longer than is recommended, she benefitted from timely diagnosis of her Heinz body anemia. This speaks to the importance of communication between the pathologist and clinical physician and the need for closely monitoring for medication induced hematologic side effects. During this phase of post‐HCT recover, medication induced complications may be overshadowed by multiple complications such as TA‐TMA, immune mediated cytopenias, or acute GVHD.

## 4 CONCLUSION

This case highlights the importance of closely monitoring medication‐induced hematologic side effects. Phenazopyridine is not a benign medication when used for prolonged periods of time and can induce hemolysis. Physicians and care providers must remain mindful of using phenazopyridine for symptomatic HC‐related dysuria.

## AUTHOR CONTRIBUTIONS

**Asmaa Ferdjallah:** Conceptualization; writing – original draft; writing – review and editing. **Susie Long:** Writing – original draft; writing – review and editing. **Vanessa Dayton:** Writing – original draft; writing – review and editing. **Ashish O. Gupta:** Writing – original draft; writing – review and editing.

## FUNDING INFORMATION

None.

## CONFLICT OF INTEREST STATEMENT

The authors declare no conflict of interest.

## CONSENT

Written informed consent was obtained from the patient to publish this report in accordance with the journal's patient consent policy.

## Data Availability

Data available on request from the authors.
